# Spontaneous breathing test in the prediction of extubation failure in the pediatric population

**DOI:** 10.1590/S1679-45082017AO3913

**Published:** 2017

**Authors:** Milena Siciliano Nascimento, Celso Moura Rebello, Luciana Assis Pires Andrade Vale, Érica Santos, Cristiane do Prado

**Affiliations:** 1 Hospital Israelita Albert Einstein, São Paulo, SP, Brazil.

**Keywords:** Respiration, artificial, Intensive care units, Child, Ventilator weaning

## Abstract

**Objective:**

To assess whether the spontaneous breathing test can predict the extubation failure in pediatric population.

**Methods:**

A prospective and observational study that evaluated data of inpatients at the Pediatric Intensive Care Unit between May 2011 and August 2013, receiving mechanical ventilation for at least 24 hours followed by extubation. The patients were classified in two groups: Test Group, with patients extubated after spontaneous breathing test, and Control Group, with patients extubated without spontaneous breathing test.

**Results:**

A total of 95 children were enrolled in the study, 71 in the Test Group and 24 in the Control Group. A direct comparison was made between the two groups regarding sex, age, mechanical ventilation time, indication to start mechanical ventilation and respiratory parameters before extubation in the Control Group, and before the spontaneous breathing test in the Test Group. There was no difference between the parameters evaluated. According to the analysis of probability of extubation failure between the two groups, the likelihood of extubation failure in the Control Group was 1,412 higher than in the Test Group, nevertheless, this range did not reach significance (p=0.706). This model was considered well-adjusted according to the Hosmer-Lemeshow test (p=0.758).

**Conclusion:**

The spontaneous breathing test was not able to predict the extubation failure in pediatric population.

## RESUMO

**Objetivo:**

Avaliar se o teste de respiração espontânea pode ser utilizado para predizer falha da extubação na população pediátrica.

**Métodos:**

Estudo prospectivo, observacional, no qual foram avaliados todos os pacientes internados no Centro de Terapia Intensiva Pediátrica, no período de maio de 2011 a agosto de 2013, que utilizaram ventilação mecânica por mais de 24 horas e que foram extubados. Os pacientes foram classificados em dois grupos: Grupo Teste, que incluiu os pacientes extubados depois do teste de respiração espontânea; e Grupo Controle, pacientes foram sem teste de respiração espontânea.

**Resultados:**

Dos 95 pacientes incluídos no estudo, 71 crianças eram do Grupo Teste e 24 eram do Grupo Controle. Os grupos foram comparados em relação a: sexo, idade, tempo de ventilação mecânica, indicação para início da ventilação mecânica e parâmetros ventilatórios pré-extubação, no Grupo Controle, e pré-realização do teste, no Grupo Teste. Não foram observadas diferenças entre os parâmetros analisados. Em relação à análise da probabilidade de falha da extubação entre os dois grupos de estudo, a chance de falha do Grupo Controle foi 1.412 maior do que a das crianças do Grupo Teste, porém este acréscimo não foi significativo (p=0,706). O modelo foi considerado bem ajustado de acordo com o teste de Hosmer-Lemeshow (p=0,758).

**Conclusão:**

O teste de respiração espontânea para a população pediátrica não foi capaz de prever a falha da extubação.

## INTRODUCTION

Invasive mechanical ventilation (IMV) is a frequently used technique at pediatric intensive care units.^1^ Approximately one out of three pediatric patients admitted to intensive care units (ICU) is estimated to require on average 4 days of respiratory support, which is currently considered a high prevalence therapeutic resource.^2^

Despite the benefits universally accepted of mechanical ventilation for children in respiratory distress, invasive support is directly associated with a series of complications. Reduction in IMV time is essential to decrease risk of complications, such as airway damage^3^ and mechanical ventilator-associated pneumonia.^4^ On the other hand, early extubation requiring reintubation is also related to some adverse outcomes, such as emergency reintubation,^5,6^ and may lead to respiratory failure and death.^7,8^ No ideal moment has been established for tracheal extubation, given its definition criteria are quite variable.^9^

Extubation refers to removal of the endotracheal cannula. Frequently considered the natural continuity of weaning, extubation has its own characteristics and predictive outcome factors that take into account, among many factors, the ability to protect airway, to manage secretions, to maintain non-obstructed upper airways.^10^ Moreover, they consider correcting the condition that led to ventilatory support, maintenance of appropriate gas exchange and respiratory muscle strength.^5,11^ Extubation failure is defined as re-insertion of the orotracheal tube within 48 hours after its removal.

Some studies in adults showed that the extubation failure rate ranges between 1.8 and 18.6%.^12,13^ Studies in the pediatric literature assessing weaning outcome predictors demonstrated the extubation failure rate varying from 16 to 22%.^14^ Recent studies have revealed serious consequences of extubation failure, including increase in mortality and morbidity rates, longer ICU and hospital stays, and high costs.^10,15^

The study of Kurachek et al.^11^ reported that 40% of patients with extubation failure had inappropriate muscle strength and pulmonary dysfunction, changes that could be recognized before extubation, by using a weaning protocol.

The spontaneous breathing test (SBT) was developed as an attempt to identify patients who are ready to discontinue ventilation, but there still are limitations for detecting who will require reintubation after undergoing a SBT. Its implementation in children can streamline weaning and decrease ventilatory support time.^10^

The present protocol aims to assess if SBT is able to predict extubation failure in the pediatric population of the pediatric ICU of *Hospital Israelita Albert Einstein*.

## OBJECTIVE

To asses if the spontaneous breathing test can be used to predict extubation failure in the pediatric population.

## METHODS

A prospective observational study that analyzed charts of patients admitted to the pediatric ICU of *Hospital Israelita Albert Einstein*, who fulfilled inclusion criteria of the sample design and required ventilatory support for a period longer than 24 hours, from May 2011 to August 2013.

Pediatric patients: who required mechanical ventilation for more than 24 hours; with resolution/control of the cause of intubation; with hemodynamic and clinical stability (afebrile; normal-for-age blood pressure, heart and respiratory rates) and without continuous sedation were included.

Study patients with one or more of the following criteria were excluded: indication for surgery in the coming 12 hours; over 18 years of age; requiring home invasive ventilatory support; primary pulmonary hypertension; diaphragm paralysis or hernia; spinal cord lesion above the lumbar region; facial trauma; cyanogenic congenital heart diseases; progressive neuromuscular disease; heart or lung transplant; tracheostomy; anatomical airway obstruction and/or post-extubation upper airway obstruction; and intracranial hypertension.

Patients who upon extubation and at end of conventional mechanical ventilation underwent SBT were compared to patients who were not submitted to the procedure, but had clinical conditions and ventilatory parameters similar to patients who were submitted to the Group test and Control.

Extubation failure, defined as need for reintubation in a period of up to 48 hours after extubation was considered the primary endpoint. The secondary endpoints considered were time on IMV, and support pressure (SP), positive end-expiratory pressure (PEEP) and oxygen concentration (Fraction of Inspired Oxygen − FiO_2_) values previous to extubation. The cases were characterized by sex, age and diagnosis that indicated mechanical ventilation.

Groups were compared as to likelihood of extubation failure. Patients were assessed and examined daily, checking as to possibility of changing the controlled ventilatory pressure mode (mode initiated by the ventilator) to breathing initiated by the patient (SP ventilatory mode or synchronized intermittent mandatory ventilation). After changing to the ventilatory mode initiated by the patient, he/she would become eligible for SBT, as of the moment the following mechanical ventilation and laboratory parameters were reached: inspiratory pressure ≤20cmH_2_O; 50% oxygen concentration FiO_2_; PEEP ≤8cmH_2_O; pH=7.35-7.45; partial carbon dioxide pressure (PaCO_2_) <50mmHg; PaO_2_/FiO_2_ ratio ≥250 and hemoglobin ≥8g/dL.

Patients scheduled for SBT had mechanical ventilator parameters adjusted as follows: PS=10cmH_2_O, PEEP=5cmH_2_O and FIO_2_<50%. Afterwards, they remained 60 minutes on PS ventilatory mode, with the parameters specified previously, with the following data collected before performing the test, and 30 and 60 minutes after beginning: vital signs, evidence of worsening of respiratory distress (intercostal or subdiaphragmatic retraction and nasal flaring) and mechanical respiratory data (tidal volume; minute-volume, respiratory rate, dynamic compliance and muscle strength) collected on the mechanical ventilator screen and capnography (end-tidal carbon dioxide − ETCO_2_). Patients were considered as having passed the SBT and fit for extubation if there were no changes in any of the parameters assessed, according to table 1. When there were changes in parameters assessed, they were not considered fit for extubation and returned to mechanical ventilation parameters previous to SBT, and were submitted to a new assessment after 24 hours.

**Table 1:** Clinical parameters to pass spontaneous breathing test

Respiratory rate	Within predicted limits
	(<50% increase in baseline values)
Tidal volume	Between 5 and 7mL/kg weight
SatO_2_	≥92%
ETCO_2_	Between 35 and 45mmHg
Heart rate	Within predicted limits (≤20% increase in baseline value)
Signs of respiratory distress*	Maximum of two signs of respiratory distress

* Sub-diaphragmatic retraction, suprasternal retraction, sternal retraction, nasal flaring.

SatO_2_: oxygen saturation; ETCO_2_: end-tidal carbon dioxide; TI: intercostal retraction.

### Statistical analysis

The distribution of numerical variables was studied for the groups set up to perform the test using histograms, asymmetry and kurtosis measurements, and Shapiro-Wilk normality tests. The Mann-Whitney non-parametric test compared groups, and data were described using medians and interquartile intervals (1^st^ quartile to 3^rd^ quartile). Sex was described by absolute frequencies and percentages, and the Pearson χ^2^ test compared groups.

Whenever needed, in order to assess the effect of the test on predicting extubation failure, a binary logistic regression model was adjusted, considering the variables that presented difference between groups, such as control variables. In this case, results were presented as odds ratios along with 95% confidence intervals, and the calibration of models was assessed by the Hosmer-Lemeshow test.

Statistical analysis was performed using the Statistical Package for the Social Sciences (SPSS) Released 2008, SPSS Statistics for Windows, version 17.0 (Chicago: SPSS Inc.), and a p<0.05 level of significance was adopted.

## RESULTS

From May 2011 to August 2013, a total of 157 children were admitted to the ICU and required orotracheal intubation. Of these, 48 were excluded because they remained on mechanical ventilation for less than 24 hours; five for having failed SBT, but extubated by medical request; eight who died or were tracheostomized; and one who had no pre-SBT pressure data registered.

Of the 95 children included in the study, 56 were male. Median age was 22 months and median mechanical ventilation time was 74 hours. Seventy-one children were extubated after SBT (Test Group) and 24 were extubated without undergoing the test (Control Group). Indications for mechanical ventilation in the Test and Control Groups were, respectively, 1 and 1 patient due to exacerbation of chronic disease (p=0.443); 18 and 3 patients due to neurological conditions (p=0.190); 20 and 8 to perform tests and/or surgical procedures (p=0.318); and 32 and 12 due to respiratory failure (p=0.765). Comparison between study groups regarding sex, age, IMV time and pre-extubation ventilatory parameters (Control Group) and pre-test (Test Group) is on table 2. No differences between parameters analyzed were observed, except for FiO_2_ (p=0.024), which presented statistical difference, however with no clinical practice relevance, namely: FiO_2_=30% in Test Group and FiO_2_=25% in Control Group.

**Table 2:** Demography of patients included in study

	Control Group	Test Group	p value
Sex			
Female	11 (45.8)	28 (39.4)	0.582
Male	13 (54.2)	43 (60.6)	
Age, months	12 (3-30)	24 (8-72)	0.074
IMV time, hours	70 (39-96)	74 (47-161)	0.206
Inspiratory pressure peak, cmH_2_O	16 (15-19)	17 (15-18)	0.516
PEEP, cmH_2_O	6 (5-6)	6 (5-6)	0.689
FiO_2_, (%)	30 (25-30)	25 (23-30)	0.024

Values as n (%) or median (interquartile interval). Shapiro-Wilk normality tests. Mann-Whitney non-parametric tests. Pearson χ^2^ Test.

IMV: invasive mechanical ventilation; PEEP: positive end expiratory pressure at end of expiration; FiO_2_: inspired oxygen fraction.

Regarding analysis of likelihood of extubation failure between the two study groups, likelihood of failure among children who did not undergo SBT was 1,412 greater than for those who did the test, although the addition was not significant (p=0.706), with a confidence interval ranging between 0.235 and 8.483. The model was considered well adjusted, according to the Hosmer-Lemeshow test (p=0.758) (Table 3).

**Table 3:** Extubation failure, according to study groups

	Extubation failure n (%)	Odds ratio	p value
Control Group (n=24)	2 (8.3)	1.412 (0.235-8.483)	0.706
Test Group (n=71)	4 (5.6)	1	

Of the 95 patients included in the study, six were re-intubated, that is, presented extubation failure, four of which in the Test Group. The cause of extubation failure was always respiratory failure; for two patients, the cause of respiratory failure was upper airway obstruction.

## DISCUSSION

The primary objective of the present study was to assess if performing SBT on the pediatric population was capable of identifying patients who were fit for extubation. Our results showed that regarding likelihood of extubation failure, it was lower among children submitted to SBT than for those who did not undergo the test, although the decrease was not significant.

Our results corroborate findings of other authors, who also found a difference between the need of reintubation presented by children submitted to SBT and the Control Group.^16,17^ Farias et al., found that SBT was able to predict extubation success in 70% of patients submitted to the test,^18^ although they concluded that SBT was not able to predict which patients required reintubation after tolerating a spontaneous breathing test.^19^ In contrast, a Brazilian study performed with 60 newborns found a significant association for extubation success in the group submitted to SBT.^20^

There was a statistically significant difference in FiO_2_ between the Test Group and Control Group, higher in the latter Group, in our study. Despite statistical significance, there was no clinical relevance for the difference, and the interquartile interval of both groups was very similar.

Regarding the impact of using SBT in mechanical ventilation time, some studies in adults showed that daily assessment associated with SBT reduces mechanical ventilation time, when compared to gradual reduction in ventilatory support.^21,22^ This current approach proposes to actively identify patients recovering from respiratory failure and capable of spontaneous breathing, possibly shortening time and complications of mechanical ventilation.^18,23^

Foronda et al., reported that performing SBT in the pediatric population was capable of reducing mechanical ventilation time in one day.^16^ In contrast, our study did not find a difference in mechanical ventilation time among children submitted to SBT and the Control Group. The difference in these findings may be related to the inclusion criteria for performing SBT. The inspiratory pressure peak of our study was 20cmH_2_O, while the literature shows values 5cmH_2_O higher, *i.e*., 25cmH_2_O. This difference may indicate that the literature performs SBT earlier, generating a greater impact on decreasing IMV time.

Unlike other studies, in which children were submitted to daily assessments to check readiness for weaning, followed by SBT for a period of 2 hours,^18^ in our study, the test was performed for a period of 1 hour. It is a protocol based on the results of Esteban et al., who compared SBT performed with a duration of 30 minutes and 120 minutes, and observed no difference in extubation failure.^24^ Moreover, most patients who failed the test already showed signs of discomfort around 35 minutes.^25^ This reduction in SBT assessment time was not associated with an increase in extubation failure rates, and we believe the drop represents a simpler way to incorporate SBT into the pediatric intensive care unit routine.

Population heterogeneity - both for indications of mechanical ventilation and age group - was one of the limitations found in the present study. The development of a prospective, randomized and controlled study is required to better assess the applicability of the test.^26,27^ Moreover, it is important to underscore the need for more in depth analysis and assessment of causes of extubation failure, such as upper airways. Given SBT is performed on intubated patients and that the main cause of extubation failure in children is upper airway obstruction, we believe that this kind of analysis can influence results.

## CONCLUSION

For patients on ventilation for more than 24 hours, in a heterogeneous pediatric population regarding some aspects, such as underlying disease and indication for mechanical ventilation, the spontaneous breathing test was not able to predict extubation failure. Changes in the spontaneous breathing test indication protocol could also change the results found in the present study.
